# Vitamin Supplementation Protects against Nanomaterial-Induced Oxidative Stress and Inflammation Damages: A Meta-Analysis of In Vitro and In Vivo Studies

**DOI:** 10.3390/nu14112214

**Published:** 2022-05-26

**Authors:** Dongli Xie, Jianchen Hu, Zhenhua Yang, Tong Wu, Wei Xu, Qingyang Meng, Kangli Cao, Xiaogang Luo

**Affiliations:** 1 College of Textile and Clothing Engineering, Soochow University, 199 Ren-Ai Road, Suzhou 215123, China; xdl202111@163.com (D.X.); hujianchen@suda.edu.cn (J.H.); 2 Department of Health and Hazards Surveillance, Shinan District Center for Disease Control and Prevention, Qingdao 266072, China; flyyzh77@163.com; 3 Shanghai Jing Rui Yang Industrial Co., Ltd., 3188 Xiupu Road, Shanghai 200122, China; wutong@lead-all.cn; 4 Shanghai Nutri-Woods Bio-Technology Co., Ltd., No. 333 Guiping Road, Shanghai 200233, China; roy@nutri-woods.com; 5 Shanghai Pechoin Daily Chemical Co., Ltd., No. 518 Anyuan Road, Shanghai 200060, China; mengqy@pechoin.com; 6 Shanghai Institute of Spacecraft Equipment, 251 Huaning Road, Shanghai 200240, China; connieckl@126.com

**Keywords:** nanomaterials, oxidative stress, inflammation, vitamin, meta-analysis

## Abstract

The extensive applications of nanomaterials have increased their toxicities to human health. As a commonly recommended health care product, vitamins have been reported to exert protective roles against nanomaterial-induced oxidative stress and inflammatory responses. However, there have been some controversial conclusions in regards to this field of research. This meta-analysis aimed to comprehensively evaluate the roles and mechanisms of vitamins for cells and animals exposed to nanomaterials. Nineteen studies (seven in vitro, eleven in vivo and one in both) were enrolled by searching PubMed, EMBASE, and Cochrane Library databases. STATA 15.0 software analysis showed vitamin E treatment could significantly decrease the levels of oxidants [reactive oxygen species (ROS), total oxidant status (TOS), malondialdehyde (MDA)], increase anti-oxidant glutathione peroxidase (GPx), suppress inflammatory mediators (tumor necrosis factor-α, interleukin-6, C-reactive protein, IgE), improve cytotoxicity (manifested by an increase in cell viability and a decrease in pro-apoptotic caspase-3 activity), and genotoxicity (represented by a reduction in the tail length). These results were less changed after subgroup analyses. Pooled analysis of in vitro studies indicated vitamin C increased cell viability and decreased ROS levels, but its anti-oxidant potential was not observed in the meta-analysis of in vivo studies. Vitamin A could decrease MDA, TOS and increase GPx, but its effects on these indicators were weaker than vitamin E. Also, the combination of vitamin A with vitamin E did not provide greater anti-oxidant effects than vitamin E alone. In summary, we suggest vitamin E alone supplementation may be a cost-effective option to prevent nanomaterial-induced injuries.

## 1. Introduction

With the rapid development of nanotechnology in the last decade, nanomaterials have been omnipresent in industrial products, medicines, food, and cosmetics due to their unique chemical and physical properties [1,2,3]. These extensive applications make nanomaterials unavoidably enter human bodies through respiratory inhalation, dermal penetration, oral ingestion, or injection routes, which subsequently results in toxic damages on various organs and tissues (e.g., lung, liver, kidney, spleen, heart, testis, et al.) and contributes to the appearance of related diseases [4,5]. Therefore, it is urgently necessary to introduce some preventive strategies to antagonize the detrimental effects of nanomaterials on human health.

Although the toxicological mechanisms of nanomaterials have not been well elucidated, activations of oxidative stress and inflammation have been demonstrated to play important roles [6,7]. An et al. used a meta-analysis to observe that titanium dioxide (TiO_2_) nanoparticle (NP) exposure significantly increased the levels of oxidants (malonaldehyde, MDA; 8-hydroxy-2-deoxyguanosine; superoxide anion; hydrogen peroxide) and reduced the levels of anti-oxidants (superoxide dismutase, SOD; glutathione, GSH; glutathione peroxidase, GPx; catalase, CAT) in murine models compared with the controls [6]. In addition, to cause redox perturbation, Moradi et al. found that TiO_2_NP gavage promoted the expression of pro-inflammatory cytokine tumor necrosis factor (TNF-α) and nuclear factor kappaB (NF-κB) in the liver tissues of rats in comparison to a control group [8]. Elevated levels of MDA, TNF-α, interleukin-6 (IL-6), C-reactive protein (CRP), and decreased levels of GSH were also revealed in the rats that received zinc oxide (ZnO) [9,10] or gold NPs [11] compared to the controls. In vitro studies showed that carbon nanotube or NP treatment inhibited the viability and induced apoptotic cell death by increasing the levels of reactive oxygen species (ROS) [12,13,14]. These findings indicate that supplementation with compounds that have anti-oxidant and anti-inflammatory functions may represent a potential strategy to prevent nanomaterial-induced toxic damages.

Currently, vitamins are one of the most commonly recommended health care products in the medical market. Several studies have reported that vitamin intake can reduce oxidative stress and inhibit pro-inflammatory responses [15,16], indicating that there are potential protective effects of vitamin supplementation against tissue injuries induced by nanomaterials. This hypothesis had been confirmed by some authors: Bayat et al. detected that relative to the ZnONP-exposed group, administration with vitamin A, C, and E significantly reduced the total oxidant status (TOS), oxidative stress index (OSI) and increased the total anti-oxidant capacity (TAC) and activities of SOD, GPx, and CAT [10], which was accompanied by decreased liver damages pathologically [10] and DNA damages [17]. Moradi et al. reported that treatment with vitamin A and E caused significant reductions in MDA, TNF-α, and NF-κB, as well as liver function index (aspartate aminotransferase, AST) [8]. However, some controversial results were also reported. The study of Afshari-Kaveh et al. showed that treatment with vitamin A alone could not prevent TiO_2_ NP-induced decreases in TAC and OSI and increases in TOS [18]. Kong et al. demonstrated that there were no significant differences in SOD, GPx, and CAT between vitamin C-treated and NP-exposed groups [19]. AL-RASHEED et al. found that vitamin E could not significantly protect liver tissues from DNA damage (indicated by a decrease in tail length) compared with ZnONP-intoxicated rats [9]. Accordingly, the protective effects of vitamin supplementation against tissue injuries induced by nanomaterials remain inconclusive.

In this study, we aimed to conduct a meta-analysis based on in vivo and in vitro studies to comprehensively evaluate whether supplementation with vitamins would enhance cell viability, inhibit cell apoptosis, and prevent tissue injuries caused by nanomaterials through exerting anti-oxidant and anti-inflammatory activities.

## 2. Materials and Methods

### 2.1. Search Strategy

This meta-analysis was performed according to the preferred reporting items for systematic reviews and meta-analysis (PRISMA). Three electronic databases, including PubMed, EMBASE and Cochrane Library, were searched from inception to January 2022 to retrieve relevant studies. The search terms used were as follows: (“nanoparticle” OR “carbon nanotube” OR “graphene”) AND (“vitamin”) AND (“animal” OR “cells”) AND (toxicity). Additionally, the reference lists of the relevant studies and reviews were also manually checked to identify potential complements.

### 2.2. Inclusion and Exclusion Criteria

Studies were included based on the participants, interventions, comparisons, outcomes, and study design (PICOS) principles: (1) participants (P): murine or murine (human) cells; (2) intervention (I): the experimental group was treated with nanomaterials and vitamins; (3) comparison (C): the control group only received nanomaterial exposure; (4) outcomes (O): cell viability, apoptosis (caspase-3), DNA damage (tail length, tail DNA %), oxidative stress (ROS, MDA, TOS, TAC, OSI, SOD, GSH, GPx, CAT; Nrf2, nuclear factor E2-related factor 2), inflammation (TNF-α, NF-κB, IL-6, CRP, IgE), global health issues (body weight) and tissue damage (such as AST; ALT, alanine aminotransferase); and (5) study design (S): controlled trials.

Articles were excluded if they met the following criteria: (1) duplicate publications; (2) non-original research, such as abstracts, case reports, reviews, letter to the editor and comments; (3) data could not be extracted or combined with other studies; and (4) irrelevant to the study objectives. Two authors independently performed the literature selection and any disagreements were resolved by discussion with a third reviewer.

### 2.3. Data Extraction

The following data were independently extracted from each qualified article by two reviewers, including the first author, the publication year, country, nanomaterial type, vitamin type, cell or animal type, number per group, dosage of nanomaterials or vitamins, duration of treatment, sample source for analysis of outcomes and data of the experimental and control groups (mean ± standard deviation). A digitizing software (Engauge Digitizer (http://digitizer.sourceforge.net/, accessed on 23 March 2022) was used to extract the data presented in the graphs. Any discrepancies in the above information were resolved by discussion with a third reviewer.

### 2.4. Quality Assessment

The Toxrtool scale was used to evaluate the quality of in vitro studies [20,21], which consisted of 18 items. A score of 1 was assigned for each item if the article satisfied the criteria; 1; otherwise, the score of 0 was given for the articles. Studies with a Toxrtool score of ≥11 points were considered to be of high quality. The SYRCLE risk-of-bias tool was used to evaluate the quality of in vivo studies [22], which consisted of ten questions about selection, performance, detection, attrition, reporting and other bias in included articles. Items were divided into ‘low’, ‘high’ or ‘unclear’ risk of bias when they were labeled as yes, no, unknown to selected articles. The methodological quality was independently evaluated by two authors. Any divergence was resolved through discussion with a third reviewer.

### 2.5. Statistical Analysis

Meta-analyses were performed using STATA 15.0 software (STATA Corporation, College Station, TX, USA). The standardized mean difference (SMD) and 95% confidence interval (CI) were calculated to assess the intervention effects of vitamins. The heterogeneity between studies was examined by Cochrane’s Q-square test and I^2^ statistic. If significant heterogeneity was present (*p* < 0.1 and I^2^ > 50%), a random-effects model was used to analyze the combined effect size; otherwise, a fixed-effects model was employed. Subgroup analyses stratified by nanomaterial types, study durations, dosages of vitamins, sample sources and animal models were conducted for indicators with at least five data analyzed to determine whether these factors influenced the results. Publication bias was evaluated by Egger’s linear regression test. The trim-and-fill method was used to adjust the pooled SMDs if a significant publication bias was detected (*p* < 0.05). The robustness of the pooled conclusions was assessed using a sensitivity analysis based on stepwise removing one study at a time.

## 3. Results

### 3.1. Study Selection

**Figure 1:**
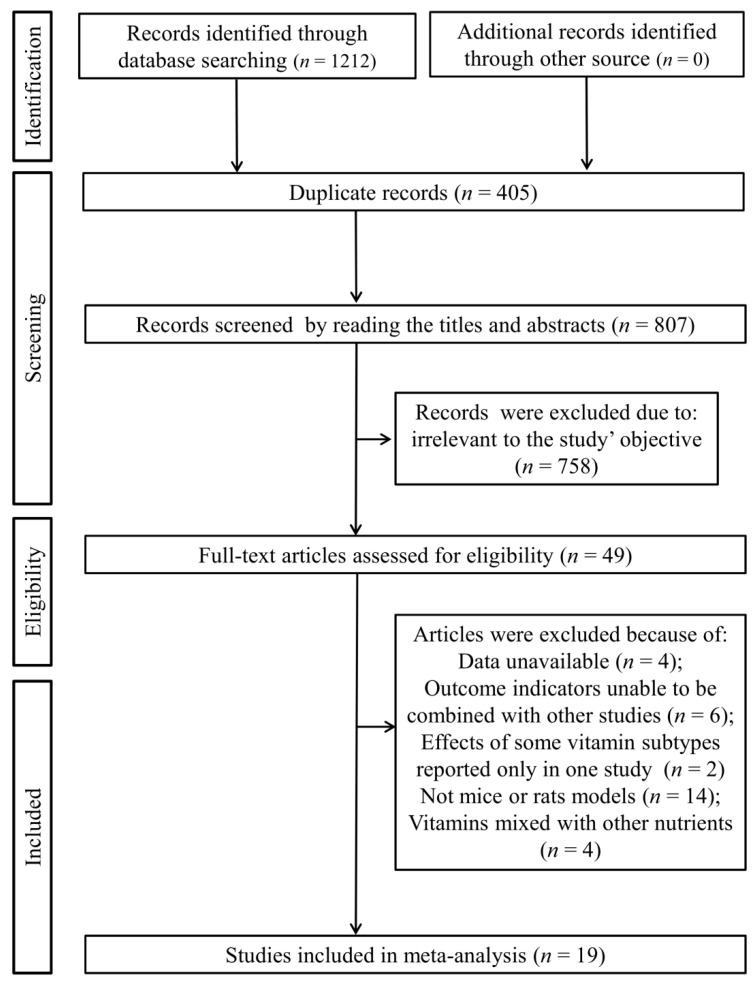
PRISMA flowchart.

A total of 1212 articles were initially retrieved through searching the electronic databases, of which 405 were duplicates. Titles and abstracts of 807 studies were reviewed and then 758 were excluded because they were irrelevant to the study’s objective. The full-texts of the remaining 49 studies were downloaded and read, after which 29 studies were eliminated for the following reasons: data were unavailable (*n* = 4); outcome indicators could not be combined with other studies (*n* = 6); the effects of some vitamin subtypes were reported only in one study (vitamin D [23] and vitamin K [24]); studies did not use mice or rats as animal models (*n* = 14); vitamins were mixed with other nutrients (*n* = 4). Finally, 19 studies were included in this meta-analysis (Figure 1) [8,9,10,11,12,13,14,17,18,19,25,26,27,28,29,30,31,32,33].

### 3.2. Study Characteristics

The basic characteristics of included articles are summarized in Table 1. Among 19 studies, seven performed in vitro experiments [12,13,14,30,31,32,33], 11 performed in vivo experiments [8,9,10,11,17,18,19,25,26,28,29] and one conducted both in vitro and in vivo experiments [27]. The in vitro studies were published between 2007 and 2019; four studies were conducted in China and the remaining four studies were, respectively, conducted in Iran, Japan, Saudi Arabia and the USA; there were four studies to, respectively, investigate the protective roles of vitamin E and vitamin C against the toxicities of various nanomaterials (including TiO_2_NPs; CoNPs, cobalt nanoparticles; AgNPs, silver nanoparticles; ZnONPs; NiONPs, nickel oxide nanoparticles; SWCNTs, single walled carbon nanotubes); the exposed cells included liver cells (human HepG2, mice liver primary cells), lung cells (human A549, rat lung epithelial cells), mouse fibroblast cells (Balb/3T3), human umbilical vein endothelial cells (HUVECs) and rat neuronal cells (PC12). The in vivo studies were published between 2013 and 2021; four studies were conducted in China, three in Iran, three in Egypt, and two in Saudi Arabia; Bayat et al. explored the protective roles of vitamin A, C and E for ZnONP-induced injuries [10]; Afshari-Kaveh et al. [18] and Moradi et al. [8] evaluated the protective roles of vitamin A, E and A + E for TiO_2_NP-induced injuries; the other eight studies only assessed the antagonized effects of vitamin E treatment for GNP-, TiO_2_NP-, ZnONP-, GO (graphene oxide)-, AgNP-induced toxicities [8,9,11,17,25,26,28,29] and one examined the effects of vitamin C for NiNP-induced toxicities [19].

### 3.3. Quality Assessment

The Toxrtool score was 16 for all studies, indicating that all in vitro studies were of high quality (Table 2). A low risk of bias was considered for the items of performance (random housing), attrition, reporting and other bias in most of animal studies except of the study of Yin et al. [26] (Table 2). Although an unclear risk of bias was assigned for most of the rest items because they were not sufficiently reported, this did not affect the determination of the indicators and the results. Thus, the quality of included in vivo studies was also acceptable.

### 3.4. Meta-Analysis for In Vitro Studies

#### 3.4.1. Effects of Vitamin E Treatment on Cell Viability

**Figure 2:**
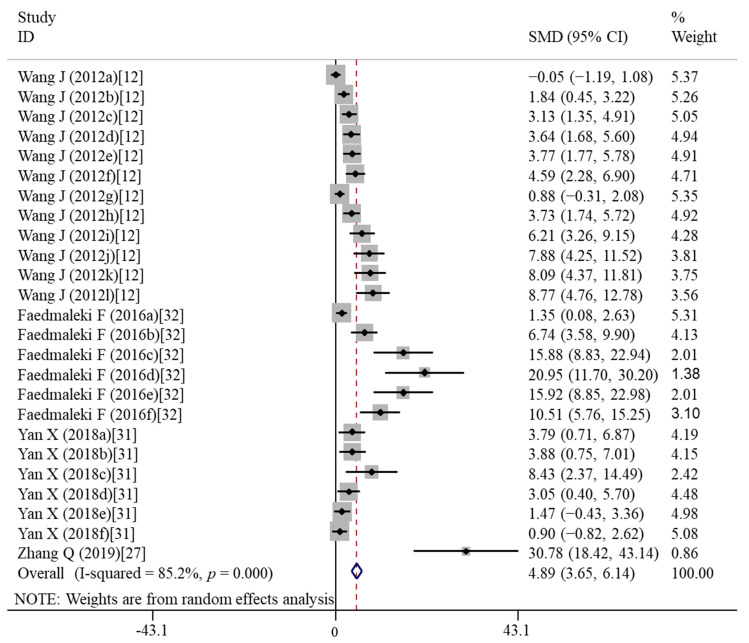
Forest plots showing the protective effects of vitamin E on the cell viability compared with the nanomaterial exposure group. a, b, c, d, e, f of the study of Wang et al. represent treatment with 10, 50, 200, 500, 1000, 2000 μM of vitamin E for 24 h; g, h, I, j, k, l of the study of Wang et al. represent treatment with 10, 50, 200, 500, 1000, 2000 μM of vitamin E for 48 h. a, b, c, d, e, f of the study of Faedmaleki et al. represent treatment with 50, 250, 500, 1000, 2500, 5000 μM of vitamin E for 24 h. a, b, c, d, e, f of the study of Yan et al. represent treatment with 2, 5, 10, 25, 50, 100 μM of vitamin E for 24 h. SMD, standardized mean difference; CI, confidence interval [12,27,31,32].

The studies of Wang et al. [12], Yan et al. [31] and Faedmaleki et al. [32] provided the results of cell viability after treatment with six dosages of vitamin E. Cells were exposed to vitamin E for 24 h and 48 h in the study of Wang et al., [12]. Only a 24-h time period was designed for other three studies [27,31,32]. Thus, 25 items of data were used for this meta-analysis. The pooled results showed that vitamin E treatment could significantly improve the cell viability compared with the nanomaterial exposure group (SMD = 4.89; 95%CI, 3.65–6.14; *p* < 0.001; I^2^ = 85.2%; *p* < 0.001) (Table 3; Figure 2). This significant effect was not changed in the subgroup analyses (Table 4).

#### 3.4.2. Effects of Vitamin E Treatment on Cell Apoptosis

**Figure 3:**
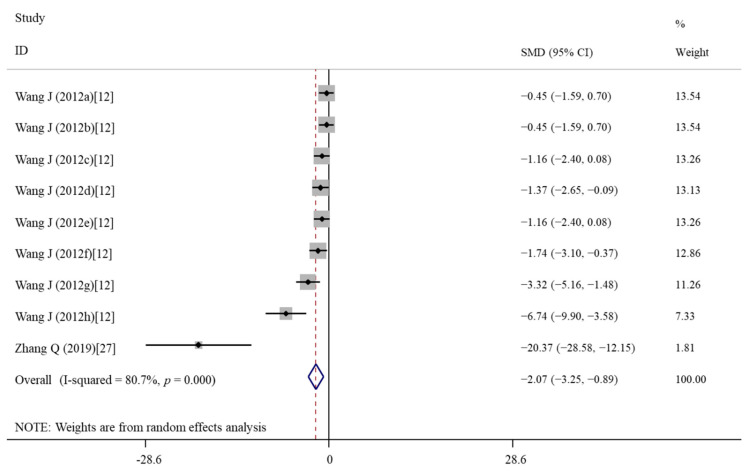
Forest plots showing the protective effects of vitamin E on Caspase-3 levels of cells compared with the nanomaterial exposure group. a, b, c, d of the study of Wang et al. represent treatment with 50, 500, 1000, 2000 μM of vitamin E for 24 h; e, f, g, h of the study of Wang et al. represent treatment with 50, 500, 1000, 2000 μM of vitamin E for 48 h. SMD, standardized mean difference; CI, confidence interval [12,27].

Caspase-3 activity was measured to represent cell apoptosis. The study of Wang et al. [12] reported the results of caspase-3 activity after treatment with four dosages (50, 500, 1000, 2000 μM) of vitamin E for 24 h and 48 h. Zhang et al. investigated the effects of vitamin E exposure (27 μM) for 24 h on the caspase-3 activity [27]. Thus, nine data were used for this meta-analysis. The pooled results showed that vitamin E treatment could significantly decrease the caspase-3 activity compared with the nanomaterial exposure group (SMD = −2.07; 95%CI, (−3.25)–(−0.89); *p* = 0.001; I^2^ = 80.7%; *p* < 0.001) (Table 3; Figure 3). The anti-apoptotic ability of vitamin E was only significant after exposure for 48 h with a dosage of ≤ 1000 μM (Table 4).

#### 3.4.3. Effects of Vitamin E Treatment on Oxidative Stress

**Figure 4:**
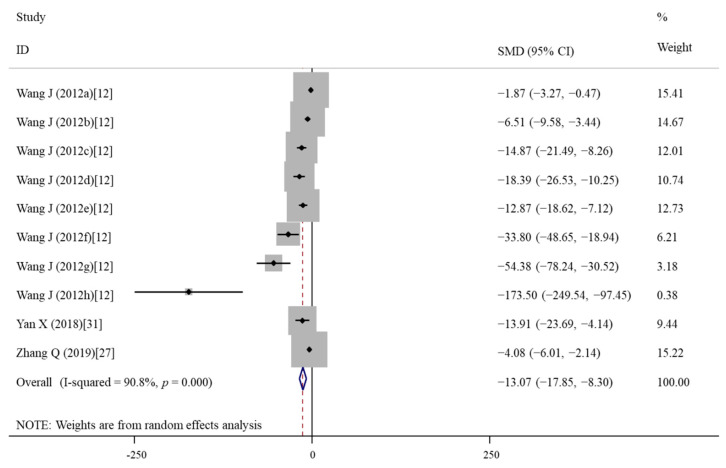
Forest plots showing the protective effects of vitamin E on ROS levels of cells compared with the nanomaterial exposure group. a, b, c, d of the study of Wang et al. represent treatment with 50, 500, 1000, 2000 μM of vitamin E for 24 h; e, f, g, h of the study of Wang et al. represent treatment with 50, 500, 1000, 2000 μM of vitamin E for 48 h. ROS, reactive oxygen species; SMD, standardized mean difference; CI, confidence interval [12,27,31].

The effects of vitamin E treatment on ROS (an indicator of oxidative stress) were evaluated in three articles [12,27,31] with ten data because four dosages (50, 500, 1000, 2000 μM) and two treatment durations were included in the study of Wang et al. [12]. The pooled results revealed that vitamin E treatment was associated with reduced ROS levels compared with the nanomaterial exposure group (SMD = −13.07; 95%CI, (−17.85)–(−8.30); *p* < 0.001; I^2^ = 90.8%; *p* < 0.001) (Table 3; Figure 4). Subgroup analyses’ results demonstrated that vitamin E treatment with a dosage of ≤1000 μM significantly inhibited the formation of ROS regardless of nanomaterials types and treatment durations (Table 4).

#### 3.4.4. Effects of Vitamin C Treatment on Cell Viability

Two studies [14,21] with four data (since three concentration gradients were designed for NiONPs in the study of Ahamed et al. [14]) investigated the effects of vitamin C treatment on the cell viability. The meta-analysis results demonstrated that vitamin C intervention could significantly increase the cell viability compared with the nanomaterial exposure group (SMD = 4.19; 95%CI, 2.37–6.01; *p* < 0.001; I^2^ = 45.3%; *p* = 0.140) (Table 3).

#### 3.4.5. Effects of Vitamin C Treatment on Oxidative Stress

Four studies [13,14,21,30] with six data (due to the three concentrations included in the study of Ahamed et al. [14]) investigated the effects of vitamin C treatment on ROS levels. The meta-analysis results demonstrated that vitamin C intervention could significantly decrease the levels of ROS compared with the nanomaterial exposure group (SMD = −6.77; 95%CI, (−12.18)–(−1.36); *p* = 0.014; I^2^ = 85.4%; *p* < 0.001) (Table 3).

### 3.5. Meta-Analysis for In Vivo studies

#### 3.5.1. Effects of Vitamin E Treatment on Body Weight

Five studies [10,17,18,26,29] with six data (including two ZnONP concentrations included in the study of Baky et al. [17]) monitored the body weight of animals after administration of vitamin E. The meta-analysis results revealed no significant differences in the body weight between vitamin E and nanomaterial exposure groups (*p* = 0.328) (Table 3). Although the subgroup analysis showed vitamin E could increase the body weight for animals exposed to AgNPs, only one study reported this effect (Table 4) and thus, the conclusion remained indefinite.

#### 3.5.2. Effects of Vitamin E Treatment on Oxidative Stress

**Figure 5:**
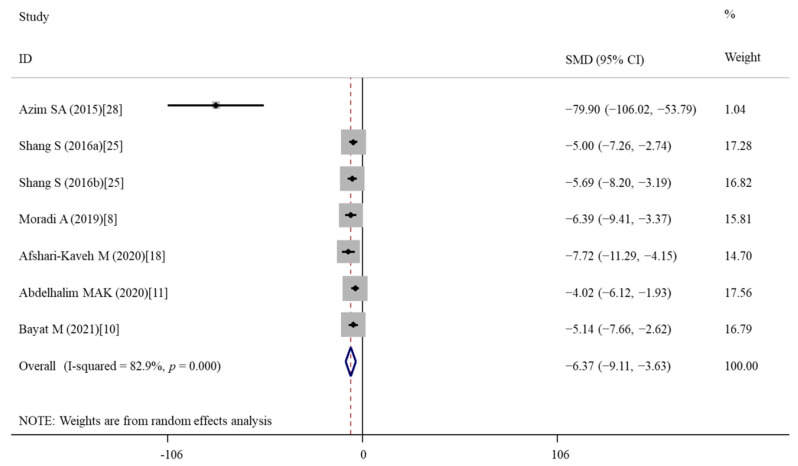
Forest plots showing the protective effects of vitamin E on MDA levels of murine models compared with the nanomaterial exposure group. a, b of the study of Shang et al. represent treatment with 0.4 and 4 mg/kg of graphene oxide. MDA, malonaldehyde; SMD, standardized mean difference; CI, confidence interval [8,10,11,18,25,28].

Six publications [8,10,11,18,25,28] with seven data (two GO concentrations included in the study of Shang et al. [25]) measured MDA levels; four articles [9,11,25,28] with six data (two concentrations included in the studies of Shang et al. [25] and AL-RASHEED et al. [9]) analyzed GSH levels; three studies [8,10,18] detected TOS, TAC, SOD and GPx; two studies examined OSI [10,18], CAT [8,10], SOD mRNA [10,18], GPx mRNA [10,18] and Nrf2 mRNA expression levels [18,28]. The summary analysis showed that the levels of pro-oxidant indicators [MDA (Figure 5): SMD = −6.37; 95%CI, (−9.11)–(−3.63); *p* < 0.001; TOS: SMD = −5.89; 95%CI, (−9.94)–(−1.84); *p* = 0.004; OSI: SMD = -4.19; 95%CI, (−5.73)–(−2.64); *p* = 0.019] were significantly decreased, while the levels of anti-oxidant indicators (TAC: SMD = 2.48; 95%CI, 1.55–3.41; *p* < 0.001; SOD: SMD = 4.19; 95%CI, 0.70–7.66; *p* = 0.019; GPx activity: SMD = 3.99; 95%CI, 2.04–5.93; *p* < 0.001; GSH: SMD = 5.26; 95%CI, 1.73–8.80; *p* = 0.004; GPx mRNA expression: SMD = 13.17; 95%CI, 1.21–25.12; *p* = 0.031) were significantly increased in the vitamin E treatment group relative to the nanomaterial exposure group (Table 3). No significant differences in the CAT activity, the mRNA expression levels of SOD and Nrf2 were present between two groups (*p* > 0.05) (Table 3). Subgroup analyses demonstrated that only vitamin E treatment for more than two weeks (regardless of dosages) significantly decreased MDA and increased GSH. The improvement effects of vitamin E on GSH may be more sensitive for mice than rats, for ZnONPs, TiO_2_NPs and GNPs than GO (Table 4).

#### 3.5.3. Effects of Vitamin E Treatment on Inflammation

**Figure 6:**
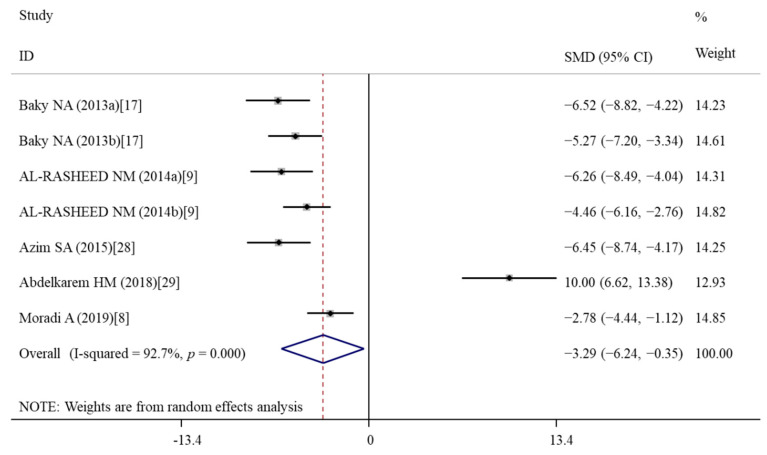
Forest plots showing the protective effects of vitamin E on TNF-α levels of murine models compared with the nanomaterial exposure group. a, b of the study of Baky et al. and AL-RASHEED et al. represent treatment with 600 and 1000 mg/kg of ZnONPs. TNF, tumor necrosis factor; SMD, standardized mean difference; CI, confidence interval [8,9,17,28,29].

**Figure 7:**
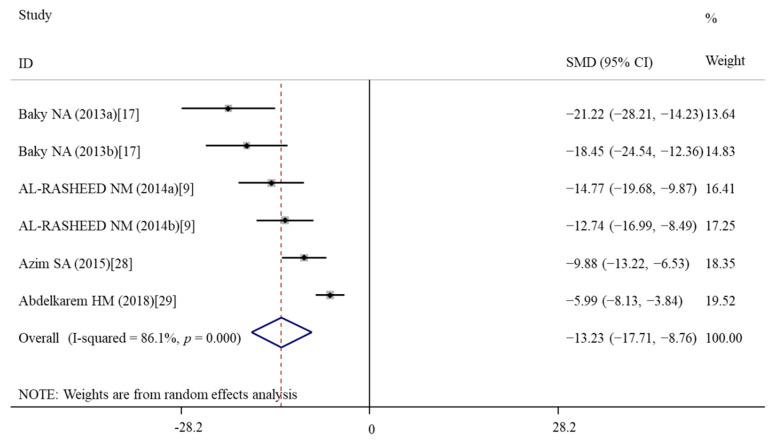
Forest plots showing the protective effects of vitamin E on IL-6 levels of murine models compared with the nanomaterial exposure group. a, b of the study of Baky et al. and AL-RASHEED et al. represent treatment with 600 and 1000 mg/kg of ZnONPs. IL, interleukin; SMD, standardized mean difference; CI, confidence interval [9,17,28,29].

Five publications [8,9,17,28,29] with seven data measured TNF-α levels; four articles [9,17,28,29] with six data collected IL-6 levels; two studies [8,10,18] with four data assessed CRP levels. The number of real data for analysis of these three inflammatory indicators was larger than the number of articles because two concentrations included in the studies of Baky et al. [17] and AL-RASHEED et al. [9]. Two studies [25,27] with three data investigated the levels of IgE. The mRNA expression level of NF-κB was analyzed in the studies of Moradi et al. [8] and Azim et al. [28]. The summary analysis showed that except of NF-κB, the levels of all other pro-inflammatory indicators were lower in the vitamin E treatment group than those in the nanomaterial exposure group [TNF-α (Figure 6): SMD = −3.29; 95%CI, (−6.24)–(−0.35); *p* = 0.028; IL-6 (Figure 7): SMD = −13.23; 95%CI, (−17.71)–(−8.76); *p* < 0.001; CRP: SMD = −5.60; 95%CI, (−6.63)–(−4.57); *p* < 0.001; IgE: SMD = −4.08; 95%CI, (−5.20)–(−2.95); *p* < 0.001]. Subgroup analyses indicated that vitamin E treatment only inhibited the production of TNF-α at the early stage (treatment for less than two weeks), but IL-6 at both of the early (≤two weeks) and later (>two weeks) stages (Table 4).

#### 3.5.4. Effects of Vitamin E Treatment on Apoptosis

**Figure 8:**
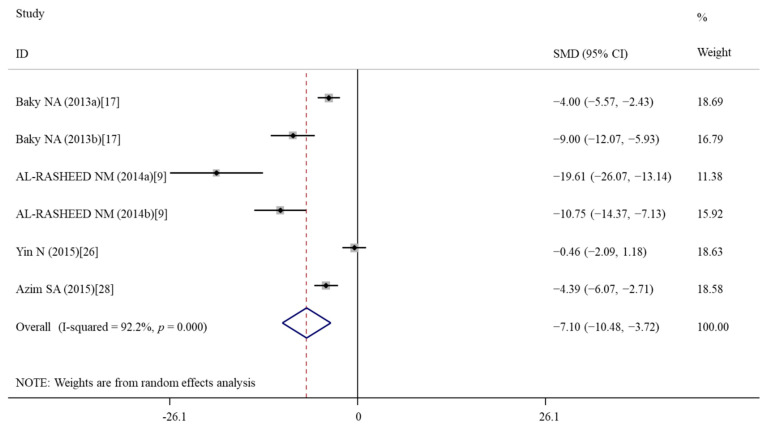
Forest plots showing the protective effects of vitamin E on Caspase-3 activity of murine models compared with the nanomaterial exposure group. a, b of the study of Baky et al. and AL-RASHEED et al. represent treatment with 600 and 1000 mg/kg of ZnONPs. SMD, standardized mean difference; CI, confidence interval [9,17,26,28].

The pooled analysis of four studies with six data [9,17,26,28] showed that compared with the nanomaterial exposure group, the caspase-3 activity was significantly decreased by vitamin E treatment (SMD = −7.10; 95%CI, (−10.49)–(−3.72); *p* < 0.001) (Table 3; Figure 8). Subgroup analyses showed that except of AgNPs, vitamin E treatment suppressed apoptosis induced by all other nanomaterials regardless of durations (Table 4).

#### 3.5.5. Effects of Vitamin E Treatment on DNA Damage

**Figure 9:**
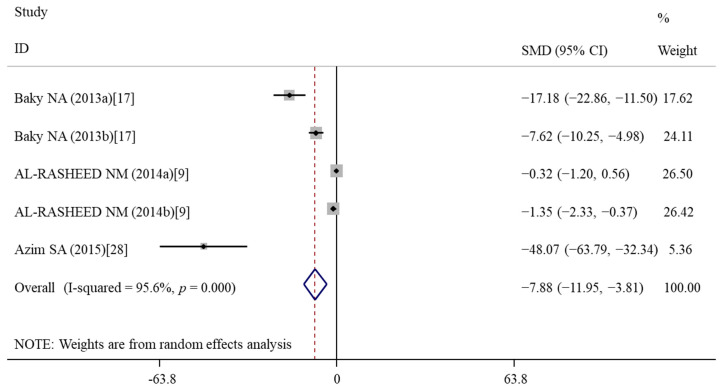
Forest plots showing the protective effects of vitamin E on tail length of murine models compared with the nanomaterial exposure group. a, b of the study of Baky et al. and AL-RASHEED et al. represent treatment with 600 and 1000 mg/kg of ZnONPs. SMD, standardized mean difference; CI, confidence interval [9,17,28].

Comet assay was performed to evaluate DNA damage for three studies [9,17,28], after which the data about the tail DNA content and the tail length were obtained. The pooled analysis of these three studies with five data revealed a significant decrease in the tail length between two groups (SMD = −7.88; 95%CI, (−11.95)–(−3.81); *p* < 0.001) (Table 3; Figure 9). There was no significant difference in the tail DNA % (*p* = 0.283). The improvement effects of vitamin E treatment on the tail length remained significant after subgroup analyses stratified by nanomaterial types and animal model types (Table 4).

#### 3.5.6. Effects of Vitamin E Treatment on Liver Function

Only liver function data (ALT, AST) could be combined for included studies [8,9,28] and thus, a meta-analysis was performed for them. The pooled analysis results showed that the level of ALT (SMD = −7.35; 95%CI, (−11.41)–(−3.29); *p* < 0.001) was significantly decreased by vitamin E treatment, but not the level of AST (Table 3).

#### 3.5.7. Effects of Vitamin C Treatment on Oxidative Stress

MDA, SOD and CAT were analyzed in two studies to explore the anti-oxidative roles of vitamin C [10,19]. Unexpectedly, the pooled analysis did not detect significant differences in these three indicators between vitamin E and nanomaterial exposure groups (*p* > 0.05) (Table 3).

#### 3.5.8. Effects of Vitamin A Treatment on Body Weight

Meta-analysis of two studies [10,18] indicated vitamin A treatment could increase the body weight of animals relative to the nanomaterial exposure group (SMD = 2.1; 95%CI, 0.06–4.14; *p* = 0.043) (Table 3).

#### 3.5.9. Effects of Vitamin A Treatment on Oxidative Stress

Meta-analysis of three studies [8,10,18] indicated vitamin A treatment could reduce the levels of MDA (SMD = −3.17; 95%CI, (−5.50)–(−0.84); *p* = 0.008) and TOS (SMD = −1.34; 95%CI, (−2.09)–(−0.59); *p* < 0.001), while increased SOD (SMD = 1.84; 95%CI, 1.01–2.67; *p* < 0.001) and GPx activity (SMD = 2.73; 95%CI, 1.77–3.7; *p* < 0.001) (Table 3). Meta-analysis of two studies [8,10] showed the activity of CAT was higher in the vitamin A treatment group relative to the nanomaterial exposure group (SMD = 3.22; 95%CI, 1.04–5.40; *p* = 0.004) (Table 3).

#### 3.5.10. Effects of Vitamin A + E Treatment on Oxidative Stress

Meta-analysis of two studies [8,18] showed that the level of MDA was reduced in the vitamin A + E treatment group compared with the nanomaterial exposure group (SMD = −8.42; 95%CI, (−11.17)–(−5.67); *p* = 0.013) (Table 3). TOS, TAC, SOD and GPx were not significantly changed (Table 3).

### 3.6. Publication Bias and Sensitivity Analysis

**Figure 10:**
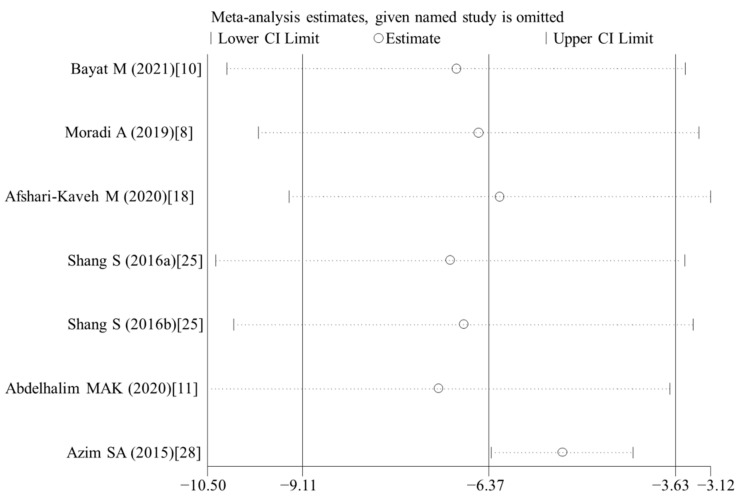
Sensitivity analysis for MDA levels of murine models treated with vitamin E. MDA, malonaldehyde; CI, confidence interval [8,10,11,18,25,28].

Egger’s test showed a publication bias existed in the analysis of several indicators with at least three data analyzed (except of the body weight, *p* = 0.081; TNF-α, *p* = 0.251 for in vivo studies with vitamin E treatment) (Table 3). However, significant results were still present for most of indicators [except of SOD (*p* = 0.435) and GSH (*p* = 0.198) in in vivo studies with vitamin E treatment, which were no longer significant after being adjusted by the trim and fill method]. Sensitivity analysis results also showed that no individual study affected the synthesized results (Figure 10).

## 4. Discussion

Although there had meta-analyses to demonstrate that vitamins can exert anti-oxidant and anti-inflammatory activities [15,16,34], no studies investigated their protective roles for nanomaterial-induced injuries until now. In addition, some meta-analysis results found the anti-oxidant and anti-inflammatory functions of vitamins were limited and even indicated vitamins exhibited potential toxic activities [35]. Thus, to prevent the hazard events induced by nanomaterials, but not cause the abuse of health care products, this study included 19 articles and performed a meta-analysis to comprehensively evaluate the roles and mechanisms of vitamins for cells and animals exposed to nanomaterials. Our meta-analysis results showed that vitamin E could antagonize nanomaterial-induced oxidative stress (mainly by reducing ROS, TOS, TAC, OSI, and MDA and increasing GPx), inflammation (significantly reducing the effects on TNF-α, IL-6, CRP, and IgE), improving cytotoxicity (manifested by an increase in the cell viability and a decrease in pro-apoptotic factor caspase-3) and genotoxicity (represented by a reduction in the tail length), which were less changed by subgroup stratifications. Pooled analysis of in vitro studies indicated that vitamin C treatment increased the cell viability and decreased ROS levels, but its anti-oxidant potential was not observed in the meta-analysis of in vivo studies. Vitamin A treatment was shown to decrease MDA (SMD: −3.17 vs. −6.37), TOS (SMD: −1.34 vs. −5.89) and increase GPx (SMD: 2.73 vs. 3.99), but its effects on these indicators seemed to be weaker than vitamin E. Also, the combination of vitamin A with vitamin E seemed not to provide greater anti-oxidant effects than vitamin E (except of MDA that was further reduced by two-fold). Accordingly, we may consider that vitamin E alone supplementation may be more cost-effective to prevent nanomaterial-induced injuries and diseases, especially for populations with occupational exposure.

Based on our results, the preventive roles of vitamin E against nanomaterial-induced injuries (apoptosis and DNA damage) were speculated to be exerted mainly through the following mechanisms: (1) as a fat-soluble vitamin, vitamin E can penetrate the lipid bilayer of the cell membrane and interact with phospholipids to stabilize bilayer structures and decrease the permeability of bilayer membranes [36], which ultimately inhibits the entrance of toxic nanomaterials into human cells [37,38]; (2) vitamin E not only quenches nanomaterial (if they are accidentally permeated to cells)-induced ROS in cell membranes [6,12,31], but also reacts with a lipid hydroperoxyl radical (LOO^•^) by donating hydrogen from its phenolic hydroxyl group at the C-6 position, resulting in the formation of lipid hydroperoxide which was subsequently transformed to non-toxic hydroxide after catalysis by GPx to terminate lipid peroxidation and decrease the levels of the end products of lipid peroxidation (MDA) [39]; (3) previous studies demonstrated that ROS induced an inflammatory response via activation of the mitogen-activated protein kinase-NF-κB signaling pathway [40,41]. Thus, the anti-inflammatory functions of vitamin E may indirectly result from its suppressive effects on oxidative stress. Furthermore, vitamin E was found to directly stimulate the production of cyclic adenosine monophosphate (cAMP) in human peripheral mononuclear cells via the EP2/EP4 receptors and adenylyl cyclase [42], which in turn activated its downstream proteins (protein kinase A and cAMP response element binding) and then suppressed the release of pro-inflammatory cytokines (such as TNF-α and IL-6) from monocytes [43]. Importantly, there was evidence to demonstrate that up-regulation of TNF-α triggered the production of IL-6 [44], while IL-6 stimulated the transcription and synthesis of CRP [45] and IgE [46]. This may be an underlying reason to explain that vitamin E suppressed the levels of TNF-α at the early stage and then IL-6 for a long time as reported in our subgroup analyses.

There are some limitations in this meta-analysis. First, the number of included in vivo and in vitro studies was still limited and the detected indicators were varying in studies, which led to less and no data pooled (such as the anti-inflammatory roles of vitamin C and A; damages on the renal, spleen, heart and brain tissues; the other vitamin types). Second, considerable heterogeneity was present among studies for the analysis of several indicators and the source of heterogeneity could not be removed by the subgroup analysis. Therefore, it is necessary to conduct more experiments on cells, animals, and humans to confirm the conclusions of our study.

## 5. Conclusions

This meta-analysis of 19 in vitro and in vivo studies provides evidence that supplementation with vitamins (especially vitamin E) may be beneficial to prevent nanomaterial-induced cytotoxicity and genotoxicity by exerting anti-oxidative and anti-inflammatory activities. Our findings support the clinical recommendations of vitamin E intake for workers with occupational exposure to nanomaterials. However, our conclusions are still needed to be confirmed by analysis of more studies of high-quality and lack of heterogeneity.

## Figures and Tables

**Table 1:** Basic characteristics of included articles.

	Author	Year	Country	Nanomaterial Type	Nanomaterial Dose	Vitamin Type	Cell (Animal) Type	Vitamin Dose	Vitamin Treatment Duration	No.	Outcomes
In vitro	Zhang Q [27]	2019	China	TiO_2_NPs	56.25 μg/mL	VE	HUVECs	27 μM	24 h	14	Cell viability, caspase 3, ROS
	Yan X [31]	2018	China	CoNPs	100 μM	VE	Balb/3T3 cells	2, 5, 10, 25, 50, 100 μM	24 h	6	Cell viability, ROS
	Faedmaleki F [32]	2016	Iran	AgNPs	121.7 μg/mL	VE	Mice liver primary cells	50, 250, 500, 1000, 2500, 5000 μM	24 h	12	Cell viability
	Liu Y [33]	2016	China	CoNPs	50 μM	VC	Balb/3T3 cells	50 μM	24 h	6	Cell viability, ROS
	Fukui H [30]	2016	Japan	ZnONPs	0.1 mg/mL	VC	A549 cells	5000 μM	6 h	6	ROS
	Ahamed M [14]	2013	Saudi Arabia	NiONPs	25 μg/mL	VC	HepG2 cells	1500 μM	24 h	6	Cell viability, ROS
	Wang J [12]	2012	China	SWCNTs	50 μg/mL	VE	PC12 cells	10, 50, 200, 500, 1000, 2000 μM	24, 48 h	12	Cell viability, caspase 3, ROS
	Sharma CS [13]	2007	USA	SWCNTs	10 μg/mL	VC	Rat lung epithelial cells	1000 μM	6 h	16	ROS
In vivo	Bayat M [10]	2021	Iran	ZnONPs	200 mg/kg	VE	Rats	100 IU/kg	3 weeks	12	Body weight, MDA, TOS, TAC, OSI, SOD, GPx, CAT
	Bayat M [10]	2021	Iran	ZnONPs	200 mg/kg	VC	Rats	100 IU/kg	3 weeks	12	MDA, SOD, CAT
	Bayat M [10]	2021	Iran	ZnONPs	200 mg/kg	VA	Rats	100 IU/kg	3 weeks	12	Body weight, MDA, TOS, TAC, OSI, SOD, GPx, CAT
	Afshari-Kaveh M [18]	2020	Iran	TiO_2_NPs	300 mg/kg	VE	Rats	100 IU/kg	3 weeks	12	Body weight, MDA, TOS, TAC, OSI, SOD, GPx, Nrf2
	Afshari-Kaveh M [18]	2020	Iran	TiO_2_NPs	300 mg/kg	VA	Rats	100 IU/kg	3 weeks	12	Body weight, MDA, TOS, TAC, OSI, SOD, GPx,
	Afshari-Kaveh M [18]	2020	Iran	TiO_2_NPs	300 mg/kg	VA + VE	Rats	100 IU/kg	3 weeks	12	MDA, TOS, TAC, SOD, GPx
	Abdelhalim MAK [11]	2020	Saudi Arabia	GNPs	50 µL	VE	Rats	200 mg/kg	1 week	12	MDA, GSH
	Moradi A [8]	2019	Iran	TiO_2_NPs	300 mg/kg	VE	Rats	100 IU/kg	2 weeks	12	MDA, TOS, TAC, SOD, GPx, CAT, NF-κB, TNF-α, ALT, AST
	Moradi A [8]	2019	Iran	TiO_2_NPs	300 mg/kg	VA	Rats	100 IU/kg	2 weeks	12	MDA, TOS, TAC, SOD, GPx, CAT
	Moradi A [8]	2019	Iran	TiO_2_NPs	300 mg/kg	VA + VE	Rats	100 IU/kg	2 weeks	12	MDA, TOS, TAC, SOD, GPx
	Zhang Q [27]	2019	China	TiO_2_NPs	500 mg/kg	VE	Mice	100 mg/kg	6 weeks	14	IgE
	Kong L [19]	2019	China	NiNPs	45 mg/kg	VC	Rats	1 g/L	2 weeks	20	MDA, SOD, CAT
	Abdelkarem HM [29]	2018	Egypt	ZnONPs	600 mg/kg	VE	Rats	200 mg/kg	3 weeks	20	Body weight, TNF-α, IL-6
	Shang S [25]	2016	China	GO	0.4, 4 mg/kg	VE	Mice	100 mg/kg	4.6 weeks	14	MDA, GSH, ROS, IgE
	Yin N [26]	2015	China	AgNPs	2 mg/kg	VE	Rats	100 mg/kg	4.6 weeks	6	Body weight, caspase-3
	Azim SA [28]	2015	Egypt	TiO_2_NPs	150 mg/kg	VE	Mice	100 mg/kg	2 weeks	20	MDA, GSH, NF-κB, Nrf2, caspase-3, tail length, DNA%,TNF-α, IL-6, ALT, AST
	AL-RASHEED NM [9]	2014	Saudi Arabia	ZnONPs	600,1000 mg/kg	VE	Rats	100 mg/kg	3 weeks	20	GSH, caspase-3, tail length, DNA%,TNF-α, IL-6, CRP, ALT
	Baky NA [17]	2013	Egypt	ZnONPs	600, 1000 mg/kg	VE	Rats	100 mg/kg	3 weeks	20	Body weight, caspase-3, tail length, DNA%, TNF-α, IL-6, CRP

TiO_2_NPs, titanium dioxide nanoparticles; ZnONPs, zinc oxide nanoparticles; CoNPs, cobalt nanoparticles; GNPs, gold nanoparticles; NiONPs, nickel oxide nanoparticles; AgNPs, silver nanoparticles; NiNPs, nickel nanoparticles; GO, graphene oxide; SWCNTs, single walled carbon nanotubes; VE, vitamin E; VA, vitamin A; VC, vitamin C; HUVECs, human umbilical vein endothelial cells; ROS, reactive oxygen species; MDA, malonaldehyde; TOS, total oxidant status; TAC, total antioxidant capacity; OSI, oxidative stress index; SOD, superoxide dismutase; GSH, glutathione; GPx, glutathione peroxidase; CAT, catalase; TNF, tumor necrosis factor; NF-κB, nuclear factor kappaB; IL, interleukin; CRP, C-reactive protein; Nrf2, nuclear factor E2-related factor 2; AST, aspartate aminotransferase; ALT, alanine aminotransferase.

**Table 2:** Quality assessments.

	Toxrtool Checklist of Study Quality																			
Reference (In Vitro)	(1)	(2)	(3)	(4)	(5)	(6)	(7)	(8)	(9)	(10)	(11)	(12)	(13)	(14)	(15)	(16)	(17)	(18)	Total	Reliability of Evidence
Zhang Q [27]	1	0	1	0	1	1	1	1	1	1	1	1	1	1	1	1	1	1	16	Reliable without restrictions
Yan X [31]	1	0	1	0	1	1	1	1	1	1	1	1	1	1	1	1	1	1	16	Reliable without restrictions
Faedmaleki F [32]	1	0	1	0	1	1	1	1	1	1	1	1	1	1	1	1	1	1	16	Reliable without restrictions
Liu Y [33]	1	0	1	0	1	1	1	1	1	1	1	1	1	1	1	1	1	1	16	Reliable without restrictions
Fukui H [30]	1	0	1	0	1	1	1	1	1	1	1	1	1	1	1	1	1	1	16	Reliable without restrictions
Ahamed M [14]	1	0	1	0	1	1	1	1	1	1	1	1	1	1	1	1	1	1	16	Reliable without restrictions
Wang J [12]	1	0	1	0	1	1	1	1	1	1	1	1	1	1	1	1	1	1	16	Reliable without restrictions
Sharma CS [13]	1	0	1	0	1	1	1	1	1	1	1	1	1	1	1	1	1	1	16	Reliable without restrictions
	**SYRCLE Checklist of Study Quality**																			
**Reference** **(In Vivo)**	**Selection Bias**						**Performance Bias**				**Detection Bias**				**Attrition Bias**		**Reporting Bias**		**Other**	
	**SG**		**BC**		**AC**		**RH**		**BI**		**ROA**		**BOA**		**IOD**		**SOR**			
Bayat M [10]	Unclear		Low		Unclear		Low		Unclear		Unclear		Unclear		Low		Low		Low	
Afshari-Kaveh M [18]	Unclear		Low		Unclear		Low		Unclear		Low		Unclear		Low		Low		Low	
Abdelhalim MAK [11]	Unclear		Unclear		Unclear		Low		Unclear		Unclear		Unclear		Low		Low		Low	
Moradi A [8]	Unclear		Unclear		Unclear		Low		Unclear		Unclear		Unclear		Low		Low		Low	
Zhang Q [27]	Unclear		Unclear		Unclear		Low		Unclear		Unclear		Unclear		Low		Low		Low	
Kong L [19]	Unclear		Unclear		Unclear		Low		Unclear		Low		Unclear		Low		Low		Low	
Abdelkarem HM [29]	Unclear		Low		Unclear		Low		Unclear		Unclear		Unclear		Low		Low		Low	
Shang S [25]	Unclear		Unclear		Unclear		Low		Unclear		Unclear		Unclear		Low		Low		Low	
Yin N [26]	Unclear		Unclear		Unclear		Unclear		Unclear		Unclear		Unclear		Low		Low		Low	
Azim SA [28]	Unclear		Unclear		Unclear		Low		Unclear		Unclear		Unclear		Low		Low		Low	
Al-Rasheed NM [9]	Unclear		Unclear		Unclear		Low		Unclear		Unclear		Unclear		Low		Low		Low	
Baky NA [17]	Unclear		Low		Unclear		Low		Unclear		Unclear		Unclear		Low		Low		Low	

(1) Test substance identification; (2) substance purity statement; (3) the source/origin information of the substance; (4) information on physicochemical properties of the test item given; (5) cell culture description; (6) the source/origin of cell culture; (7) necessary information on cell culture properties, conditions of cultivation and maintenance; (8) the method of vitamin administration; (9) doses or concentration statement; (10) frequency and duration of exposure as well as time-points of observations statement; (11) have negative controls; (12) have positive controls; (13) the number of replicates; (14) are the study endpoint(s) and their method(s) of determination clearly described?; (15) is the description of the study results for all endpoints investigated transparent and complete?; (16) are the statistical methods for data analysis given and applied in a transparent manner?; (17) is the study design chosen appropriate for obtaining the substance-specific data aimed at?; (18) are the quantitative study results reliable? Each item of Toxrtool obtained a score of 1 if the article satisfied the criteria; 1; otherwise, the score of 0 was given for the articles. SG, sequence generation; BC, baseline characteristics; AC, allocation concealment; RH, random housing; BI, blinding of investigators; ROA, random outcome assessment; BOA, blinding of outcome assessor; IOD, incomplete outcome data; SOR, selective outcome reporting.

**Table 3:** Meta-analysis results.

Studies	No.	SMD	95%CI	*p*_E_-Value	I^2^	*p*_H_-Value	Model	Egger
VE (in vitro)								
Cell viability	25	4.89	3.65, 6.14	**<0.001**	85.2	<0.001	R	<0.001
Caspase-3	9	−2.07	−3.25, −0.89	**0.001**	80.7	<0.001	R	<0.001
ROS	10	−13.07	−17.85, −8.30	**<0.001**	90.8	<0.001	R	<0.001
VC (in vitro)								
Cell viability	4	4.19	2.37, 6.01	**<0.001**	45.3	0.140	F	<0.001
ROS	6	−6.77	−12.18, −1.36	**0.014**	85.4	<0.001	R	0.002
VE (in vivo)								
Body weight	6	0.52	−0.52, 1.56	0.328	78.8	<0.001	R	0.081
MDA	7	−6.37	−9.11, −3.63	**<0.001**	82.9	<0.001	R	<0.001
TOS	3	−5.89	−9.94, −1.84	**0.004**	84.1	0.002	R	0.006
TAC	3	2.48	1.55, 3.41	**<0.001**	34.5	0.217	F	0.035
OSI	2	−4.19	−5.73, −2.64	**<0.001**	9.2	0.294	F	-
SOD activity	3	4.19	0.70, 7.66	**0.019**	86.0	0.001	R	0.016
GPx activity	3	3.99	2.04, 5.93	**<0.001**	58.8	0.088	R	0.023
CAT activity	2	17.33	−9.19, 43.85	0.200	92.9	<0.001	R	-
GSH	6	5.26	1.73, 8.80	**0.004**	93.3	0.000	R	0.004
SOD mRNA	2	7.52	−2.96, 18.00	0.159	91.5	0.001	R	-
GPx mRNA	2	13.17	1.21, 25.12	**0.031**	84.3	0.012	R	-
Nrf2 mRNA	2	−27.48	−88.49, 33.54	0.377	97.4	<0.001	R	-
TNF-α	7	−3.29	−6.24, −0.35	**0.028**	92.7	<0.001	R	0.251
IL-6	6	−13.23	−17.71, −8.76	**<0.001**	86.1	<0.001	R	<0.001
CRP	4	−5.60	−6.63, −4.57	**<0.001**	30.7	0.228	F	<0.001
IgE	3	−4.08	−5.20, −2.95	**<0.001**	0.0	0.631	F	0.009
NF-κB mRNA	2	−26.00	−57.03, 5.02	0.100	94.4	<0.001	R	-
Caspase-3	6	−7.10	−10.49, −3.72	**<0.001**	92.2	<0.001	R	0.019
Tail length	5	−7.88	−11.95, −3.81	**<0.001**	95.6	<0.001	R	0.001
Tail DNA %	5	−0.94	−2.66, 0.78	0.283	90.5	<0.001	R	0.006
ALT	4	−7.35	−11.41, −3.29	**<0.001**	90.7	<0.001	R	0.021
AST	2	−14.61	−35.90, 6.69	0.179	95.8	<0.001	R	-
VC (in vivo)								
MDA	2	−1.49	−4.13, 1.15	0.268	86.7	0.006	R	-
SOD	2	2.70	−4.04, 9.43	0.433	94.7	<0.001	R	-
CAT	2	10.17	−10.17, 30.50	0.327	94.7	<0.001	R	-
VA (in vivo)								
Body weight	2	2.10	0.06, 4.14	**0.043**	71.1	0.063	R	-
MDA	3	−3.17	−5.50, −0.84	**0.008**	78.6	0.009	R	0.050
TOS	3	−1.34	−2.09, −0.59	**<0.001**	16.6	0.302	F	<0.001
TAC	3	1.75	−0.25, 3.75	0.086	81.8	0.004	R	0.038
SOD	3	1.84	1.01, 2.67	**<0.001**	42.2	0.177	F	<0.001
GPx	3	2.73	1.77, 3.70	**<0.001**	0.0	0.441	F	0.008
OSI	2	−3.41	−8.63, 1.80	0.200	90.6	0.001	R	-
CAT	2	3.22	1.04, 5.40	**0.004**	61.6	0.107	R	-
SOD mRNA	2	9.75	−4.80, 24.31	0.189	92.4	<0.001	R	-
GPx mRNA	2	13.32	−4.24, 30.87	0.137	91.2	0.001	R	-
VA + VE (in vivo)								
MDA	2	−8.42	−11.17, −5.67	**0.013**	0.0	0.461	F	-
TOS	2	−3.90	−10.54, 2.75	0.250	92.4	<0.001	R	-
TAC	2	2.00	−0.01, 4.01	0.051	71.4	0.062	R	-
SOD	2	2.45	−0.29, 5.20	0.080	80.6	0.023	R	-
GPx	2	3.67	−0.79, 8.12	0.107	87.2	0.005	R	-

ROS, reactive oxygen species; MDA, malonaldehyde; TOS, total oxidant status; TAC, total antioxidant capacity; OSI, oxidative stress index; SOD, superoxide dismutase; GSH, glutathione; GPx, glutathione peroxidase; CAT, catalase; TNF, tumor necrosis factor; NF-κB, nuclear factor kappaB; IL, interleukin; CRP, C-reactive protein; Nrf2, nuclear factor E2-related factor 2; AST, aspartate aminotransferase; ALT, alanine aminotransferase; SMD, standardized mean difference; CI, confidence interval; F, fixed-effects; R, random-effects; *p*_H_-value, significance for heterogeneity; *p*_E_-value, significance for treatment effects. Bold indicated the outcomes significantly changed by vitamins.

**Table 4:** Subgroup analysis.

Studies		No.	SMD	95%CI	*p*_E_-Value	I^2^	*p*_H_-Value	Model
VE (in vitro)								
Cell viability								
Nanomaterials type	TiO_2_NPs	1	30.78	18.42, 43.14	**<0.001**	-	-	R
	SWCNTs	12	3.89	2.48, 5.30	**<0.001**	83.8	<0.001	R
	AgNPs	6	11.03	5.23, 16.83	**<0.001**	91.8	<0.001	R
	CoNPs	6	2.64	1.15, 4.12	**<0.001**	46.6	0.096	R
Duration	24 h	19	4.68	3.24, 6.13	**<0.001**	85.1	<0.001	R
	48 h	6	5.63	2.73, 8.52	**<0.001**	87.2	<0.001	R
Dosage	≤1000 μM	21	4.15	2.92, 5.38	**<0.001**	83.8	<0.001	R
	>1000 μM	4	9.18	4.82, 13.53	**<0.001**	77.2	0.004	R
Caspase-3								
Nanomaterials type	TiO_2_NPs	1	−20.37	−28.58, −12.15	**<0.001**	-	-	R
	SWCNTs	8	−1.58	−2.43, −0.72	**<0.001**	66.2	0.004	R
Duration	24 h	5	−1.51	−3.12, 0.10	0.066	82.9	<0.001	R
	48 h	4	−2.78	−4.53, −1.03	**0.002**	75.9	0.006	R
Dosage	≤1000 μM	7	−1.73	−2.97, −0.46	**0.008**	79.9	<0.001	R
	>1000 μM	2	−3.85	−9.10, 1.39	0.150	89.5	0.002	R
ROS								
Nanomaterials type	TiO_2_NPs	1	−4.08	−6.02, −2.14	**<0.001**	-	-	R
	SWCNTs	8	−17.07	−24.40, −9.75	**<0.001**	92.6	<0.001	R
	CoNPs	1	−13.91	−17.85, −8.30	**0.005**	-	-	R
Duration	24 h	6	−8.02	−11.75, −4.30	**<0.001**	86.7	<0.001	R
	48 h	4	−46.94	−76.37, −17.51	**0.002**	90.7	<0.001	R
Dosage	≤1000 μM	8	−10.95	−15.34, −6.55	**<0.001**	89.6	<0.001	R
	>1000 μM	2	−91.15	−242.86, 60.57	0.239	93.7	<0.001	R
VE (in vivo)								
Body weight								
Nanomaterials type	ZnONPs	4	0.07	−1.08, 1.23	0.901	80.7	0.001	R
	TiO_2_NPs	1	1.04	−0.18, 2.26	0.095	-	-	R
	AgNPs	1	3.04	0.40, 5.69	**0.024**	-	-	R
Dosage	≤100 mg/kg	5	0.81	−0.48, 2.11	0.220	81.0	<0.001	R
	>100 mg/kg	1	0.52	−0.51, −1.40	0.267	-	-	R
MDA								
Nanomaterials type	ZnONPs	1	−5.14	−7.66, −2.62	**<0.001**	-	-	R
	TiO_2_NPs	3	−17.00	−28.58, −5.42	**0.004**	93.3	<0.001	R
	GNPs	1	−4.03	−6.12, −1.93	**<0.001**	-	-	R
	GO	2	−5.31	−6.99, −3.63	**<0.001**	0.0	0.688	F
Dosage	≤100 mg/kg	6	−7.11	−10.50, −3.71	**<0.001**	84.8	<0.001	R
	>100 mg/kg	1	−4.03	−6.12, −1.93	**<0.001**	-	-	R
Duration	≤2 weeks	2	−41.95	−113.95, −30.05	0.253	96.7	<0.001	R
	>2 weeks	5	−5.15	−6.26, −4.05	**<0.001**	0.0	0.509	F
Sample source	Liver	3	−14.14	−24.25, −4.03	**0.006**	93.6	<0.001	R
	Spleen	1	−7.72	−11.29, −4.16	**<0.001**	-	-	R
	Lung	2	−5.31	−6.99, −3.63	**<0.001**	0.0	0.688	F
	Kidney	1	−4.03	−6.12, −1.93	**<0.001**	-	-	R
Animal model	Mice	3	−12.17	−20.98, −3.37	**0.007**	93.6	<0.001	R
	Rats	4	−5.39	−6.88, −3.90	**<0.001**	19.1	0.295	F
GSH								
Nanomaterials type	ZnONPs	2	8.54	3.04, 14.04	**0.002**	83.9	0.013	R
	TiO_2_NPs	1	6.96	4.52, 9.39	**<0.001**	-	-	R
	GNPs	1	3075.00	1727.35, 4422.65	**<0.001**	-	-	R
	GO	2	1.46	−0.23, 3.16	0.090	72.3	0.057	R
Dosage	≤100 mg/kg	5	5.16	2.10, 8.22	**0.001**	92.7	<0.001	R
	>100 mg/kg	1	3075.00	1727.35, 4422.65	**<0.001**	-	-	R
Duration	≤2 weeks	2	1463.89	−1.5 × 10^3^, 4465.95	0.339	95.0	<0.001	R
	>2 weeks	4	4.69	1.38, 8.01	**0.005**	92.9	<0.001	R
Sample source	Liver	3	7.74	4.97, 10.52	**<0.001**	67.9	0.044	R
	Lung	2	1.46	−0.23, 3.16	0.090	72.3	0.057	R
	Kidney	1	3075.00	1727.35, 4422.65	**<0.001**	-	-	R
Animal model	Mice	3	3.14	0.18, 6.10	**0.037**	90.9	<0.001	R
	Rats	3	5.26	8.93, −2.18	0.115	92.3	<0.001	R
TNF-α								
Nanomaterials type	ZnONPs	5	−2.68	−6.96, 1.60	0.219	94.7	<0.001	R
	TiO_2_NPs	2	−4.53	−8.13, −0.93	**0.014**	84.6	0.011	R
Dosage	≤100 mg/kg	6	−5.14	−6.39, −3.90	**<0.001**	57.5	0.038	R
	>100 mg/kg	1	10.00	6.62, 13.38	**<0.001**	-	-	R
Duration	≤2 weeks	2	−4.53	−8.13, −0.93	**0.014**	84.6	0.011	R
	>2 weeks	5	−2.68	−6.96, 1.60	0.219	94.7	<0.001	R
Sample source	Liver	2	−4.53	−8.13, −0.93	**0.014**	84.6	0.011	R
	Serum	5	−2.68	−6.96, 1.60	0.219	94.7	<0.001	R
Animal model	Mice	1	−6.45	−8.74, −4.17	**<0.001**	-	-	R
	Rats	6	−2.75	−6.10, 0.60	0.108	93.6	<0.001	R
IL-6								
Nanomaterials type	ZnONPs	5	−13.23	−14.18, −20.08	**<0.001**	88.9	<0.001	R
	TiO_2_NPs	1	−9.88	−13.22, −6.53	**<0.001**	-	-	R
Dosage	≤100 mg/kg	5	−14.71	−18.50, −10.91	**<0.001**	67.0	0.017	R
	>100 mg/kg	1	−5.99	−8.13, −3.85	**<0.001**	-	-	R
Duration	≤2 weeks	1	−9.88	−13.22, −6.53	**<0.001**	-	-	R
	>2 weeks	5	−13.23	−14.18, −20.08	**<0.001**	88.9	<0.001	R
Sample source	Liver	5	−13.23	−14.18, −20.08	**<0.001**	88.9	<0.001	R
	Serum	1	−9.88	−13.22, −6.53	**<0.001**	-	-	R
Animal model	Mice	1	−9.88	−13.22, −6.53	**<0.001**	-	-	R
	Rats	5	−13.23	−14.18, −20.08	**<0.001**	88.9	<0.001	R
Caspase-3								
Nanomaterials type	ZnONPs	4	−10.17	−10.45, −4.90	**<0.001**	91.0	<0.001	
	TiO_2_NPs	1	−4.39	−6.07, −2.71	**<0.001**	-	-	
	AgNPs	1	−0.46	−2.09, 1.18	0.584	-	-	
Duration	≤2 weeks	1	−4.39	−6.07, −2.71	**<0.001**	-	-	
	>2 weeks	5	−8.01	−12.55, −3.47	**0.001**	93.8	<0.001	
Sample source	Liver	3	−11.01	−18.55, −3.48	**0.004**	92.6	<0.001	
	Brain	1	−0.46	−2.09, 1.18	0.584	-	-	
	Heart	2	−6.32	−11.21, −1.43	**0.011**	87.6	0.004	
Animal model	Mice	1	−4.39	−6.07, −2.71	**<0.001**	-	-	
	Rats	5	−8.01	−12.55, −3.47	**0.001**	93.8	<0.001	
Tail length								
Nanomaterials type	ZnONPs	4	−5.21	−8.60, −1.82	**0.003**	94.7	<0.001	R
	TiO_2_NPs	1	−48.07	−63.79, −32.34	**<0.001**	-	-	R
Sample source	Liver	3	−3.35	−7.19, 0.50	0.088	94.6	<0.001	R
	Heart	2	−12.05	−21.40, −2.70	**0.012**	88.8	0.003	R
Animal model	Mice	1	−48.07	−63.79, −32.34	**<0.001**	-	-	R
	Rats	4	−5.21	−8.60, −1.82	**0.003**	94.7	<0.001	R
Tail DNA %								
Nanomaterials type	ZnONPs	4	−0.73	−1.42, −0.05	**0.035**	53.4	<0.092	R
	TiO_2_NPs	1	−166.00	−220.23, −111.77	**<0.001**	-	-	R
Sample source	Liver	3	−1.11	−4.79, 2.58	0.557	94.4	<0.001	R
	Heart	2	−1.19	−2.57, 0.18	0.089	74.1	0.050	R
Animal model	Mice	1	−166.00	−220.23, −111.77	**<0.001**	-	-	R
	Rats	4	−0.73	−1.42, −0.05	**0.035**	53.4	<0.092	R

ROS, reactive oxygen species; MDA, malonaldehyde; TNF, tumor necrosis factor; IL, interleukin; TiO_2_NPs, titanium dioxide nanoparticles; ZnONPs, zinc oxide nanoparticles; CoNPs, cobalt nanoparticles; GNPs, gold nanoparticles; NiONPs, nickel oxide nanoparticles; AgNPs, silver nanoparticles; GO, graphene oxide; SWCNTs, single walled carbon nanotubes; SMD, standardized mean difference; CI, confidence interval; F, fixed-effects; R, random-effects; *p*_H_-value, significance for heterogeneity; *p*_E_-value, significance for treatment effects. Bold indicated the outcomes significantly changed by vitamins.

## Data Availability

All data can be obtained in published articles.
